# Glucose Variability Analysis in Two Large-Scale and Real-World Data Sets of Open-Source Automated Insulin Delivery Systems

**DOI:** 10.1177/19322968231198871

**Published:** 2023-09-26

**Authors:** Drew Cooper, Bernd Reinhold, Arsalan Shahid, Dana M. Lewis

**Affiliations:** 1 Institute of Medical Informatics, Charité—Universitätsmedizin Berlin, Berlin, Germany; 2 Eddimed, Stuttgart, Germany; 3 CeADAR, Ireland’s Centre for Applied AI, University College Dublin, Dublin, Ireland; 4 OpenAPS, Seattle, WA, USA

**Keywords:** type 1 diabetes, automated insulin delivery, glycemic variability, machine learning, CGM, glucose

## Abstract

**Background:**

Open-source automated insulin delivery (OS-AID) systems combine commercially available insulin pumps and continuous glucose monitors with open-source algorithms to automate insulin dosing for people with insulin-requiring diabetes. Two data sets (OPEN and the OpenAPS Data Commons) contain anonymized OS-AID user data.

**Methods:**

We assessed glycemic variability (GV) outcomes in the OPEN data set and characterized it alongside a comparison to the n = 122 version of the OpenAPS Data Commons. Glucose data are analyzed using an unsupervised machine learning algorithm for clustering, and GV metrics are quantified using statistical tests for distribution comparison. Demographic data are also analyzed quantitatively.

**Results:**

The n = 75 OPEN data set contains 36 827 days worth of data. Mean TIR is 82.08% (TOR < 70: 3.66%; TOR > 180: 14.3%). LBGI (*P* < .05) differs by gender whereas HBGI distributions are similar (*P* > .05). GV metrics (except TOR < 70, LBGI) show a statistically significant difference (*P* < .05) between data sets.

**Conclusions:**

Both the OPEN and OpenAPS Data Commons data sets show TOR < 70, TIR, and TOR > 180 within recommended goals, adding additional evidence of real-world efficacy of OS-AID. Future research should evaluate in more detail potential data set differences and relationships between individual patterns of user behaviors and GV outcomes.

## Introduction

Open-source automated insulin delivery (OS-AID) systems combine commercially available insulin pumps and continuous glucose monitors with open-source algorithms to automate insulin dosing based on glucose levels.^1^ One OS-AID has multiple randomized control trials recently published demonstrating safety and efficacy;^2-4^ another OS-AID system was recently cleared by the US Food and Drugs Administration (FDA).^5^

Data from OS-AID generate a wealth of information, including continuous glucose monitor (CGM) data, user-entered information (such as the amount of carbohydrates consumed or target changes made) with user-unique settings (such as basal rates, insulin sensitivity factor, and carbohydrate ratio), and algorithm generated-data such as predicted glucose values and novel calculated variables (eg, autosensitivity,^6^ a method for detecting changes to insulin sensitivity).

Two online repositories exist on the Open Humans platform^7^ to facilitate anonymous contributions of diabetes-related data for research. The OpenAPS Data Commons collects data from all types of OS-AID users^8^ with more than 46 070 days worth of CGM data alongside additional data from insulin pumps, CGM data, user-entered data, and demographics information.^9^ The OPEN project also established a repository on Open Humans,^10,11^ and n = 129 OPEN Survey participants also chose to donate diabetes device data, of which n = 96 were OS-AID users.

Previous work done by Shahid and Lewis evaluated the n = 122 version of the OpenAPS Data Commons with a demographic analysis, glucose profile clustering, gender-based analysis of glycemic variability, and a time series analysis based on gender.^9^

This paper assesses glycemic variability in the OPEN data set and characterizes it in a comparison to the OpenAPS Data Commons data set.

## Methods

### Data Collection

Demographic data were collected from OPEN Survey participants through the REDCap platform.^12^ Data management was carried out using an open-source web portal,^13^ and diabetes data were donated on Open Humans.

### Identification of Unique Participants

Data sets were first evaluated for potential duplicates. The OPEN data set was examined for AID users or caregivers of children using AID who had demographic information from the OPEN Survey; had uploaded data; and had not withdrawn. This list (n = 96) was then checked for duplicates against the OpenAPS Data Commons.

### Deduplication Based on Glucose Data

Because each data set has randomly generated project identification numbers that are distinct to each project, we used glucose data and timestamps to identify potential duplicates between the two data sets.

**Figure 1.**
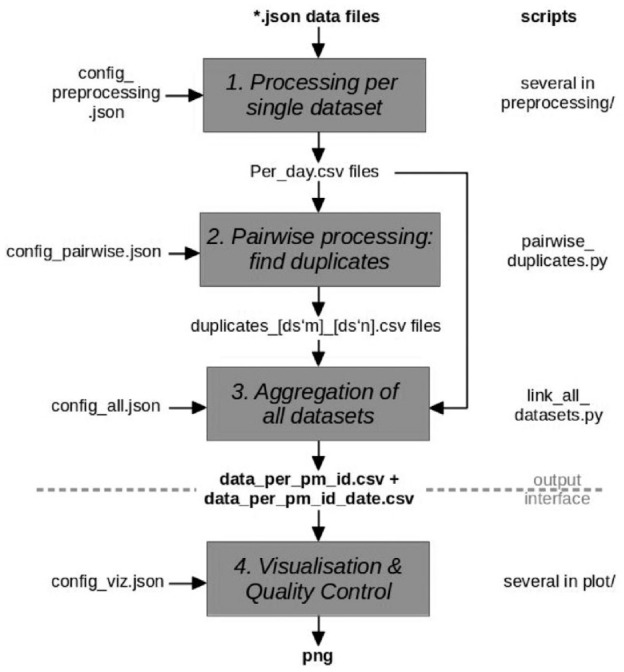
Processing pipeline for deduplication of extracted json data files with glucose data. The resulting output provides a mapping of Open Humans project member IDs of participants used in different data sets. If two project member IDs have been found to be belonging to the same person, both project member IDs show up in the same row. A second file contains the same project member ID mapping, and additionally associating them with the dates present in the respective data sets.

Figure 1 shows the flow of data through the various processing scripts, which are available open source on GitHub,^14^ and Supplementary Figure 1 displays the formula for defining the symmetric differences between individuals.

### Final Selection of Participants

Supplementary Figure 2 outlines the processes for excluding individuals who were not AID users, removing 21 duplicates that were already present and analyzed in the OpenAPS Data Commons, and eliminating those who did not appear to have AID data within their donated data set. The final number of participants in the OPEN data set used in the subsequent analyses and comparisons is n = 75.

### Analyses

Analysis of OPEN data was performed using modified open-source processing scripts^9,15^ to clean glucose data. Values within the range of 400 to 1000 mg/dL were capped at 400 and values above 1000 were omitted. The glucose data were analyzed using the accompanying timestamps, which is normalized to the user’s local time zone at the point of data recording.

The following glucose characteristics and statistics were analyzed: time-in-range (TIR), time-out-of-range (TOR), high and low blood glucose index (HBGI/LBGI), coefficient of variation (CV), counts, mean, standard deviation (SD), minimum, maximum, interquartile range (IQR), as well as multiple statistical tests, including z-tests for comparing distributions, when continuous glucose data were recorded.

An unsupervised learning approach—utilizing an agglomerative clustering algorithm—was also applied to determine if there were distinct patterns in glucose data profiles of the group of insulin-requiring individuals using open-source AID.

Results from the OPEN data set were then compared to the previous results from the OpenAPS Data Commons.

## Results

### Summary Statistics

The cleaned OPEN data set consists of data from 36 827 days from n = 75 participants. On average, participants had 491 days of data and their glucose levels had an average mean of 132.20 mg/dL and average SD of 43.34 mg/dL. Supplementary Figure 3 displays a histogram highlighting participant clusters by glucose data duration, notably at intervals of 10 days, 100 to 200 days, and near 1000 days.

Demographic data for these participants are listed in Supplementary Table 1. The average age was 41 years of age (minimum 5; maximum 78), with seven participants being children (<18 years of age). Of those who reported gender (n = 1 did not report and was not included in the gender sub-analyses), n = 47 are male and n = 27 are female. Participants were from 18 different countries; most were from Germany (n = 33), the United States (n = 11), and the United Kingdom (n = 6), and the majority (n = 68) of participants who reported ethnicity were white. The majority of participants (n = 73) reported having type 1 diabetes; one reported having type 2 diabetes. On average at the time of the OPEN survey, participants had been living with diabetes for 25 years (minimum 3 years, maximum 55 years), had 11.88 years of experience using an insulin pump (minimum < 1 year; maximum 38 years), and had 4.59 years of CGM (minimum < 1 year; maximum 12 years) experience.

The majority of participants in this data set at the time of the survey reported using AndroidAPS (n = 55), Loop (n = 22), and OpenAPS (n = 4) OS-AID systems. The average length of closed loop usage was 1.67 years (minimum < 1 year; maximum 4 years) experience. Supplementary Figure 4 visualizes mean glucose and SD ranges.

### Glucose Data Analysis

Supplementary Figure 5 illustrates the distribution of glucose TIR and TOR in percentages in the OPEN data set. The mean TIR (and TOR < 70, TOR > 180) in the OPEN data set is 82.08% (TOR < 70: 3.66%; TOR > 180: 14.3%). An examination of the distribution of TOR < 70 and TOR > 180 within the data set reveals a greater tendency for individuals to have values of TOR > 180, as compared to those who have values of TOR < 70.

Table 1 presents a summary of statistical measures including minimum, maximum, mean, quartiles (Q1, Q2, and Q3), and IQRs for each glucose analysis and variability metric. Salient results include the following:

1. The minimum, mean, and maximum of interday glucose SD and CV is {23, 43, 76} mg/dL and {20, 32, 50}%, respectively. The IQR is a robust measure of variability representing the range of middle 50% of the data and is calculated as a difference between Q3 and Q1. SD and CV have IQR values of 14.97 and 6.05, respectively.
2. The glucose rate of change (ROC) per minute for each individual is computed using the following formula: $\left[glucose\;(t_{i})-glucose\;(t_{i-1})/(t_{i}-t_{i-1})\right]$ . The standard deviation of ROC (SD ROC) has a minimum, mean, and maximum of {0.76, 1.29, 2.29} mg/dL per minute.
3. Mean TIR in the OPEN data set is 82.08%. Fewer than 25% of the individuals exhibit TIR values less than 76.9%. 25% demonstrate TIR values above 89.8%. The min, mean, and max for TOR < 70 and TOR > 180 are {0.05, 3.66, 14.67}% and {0.83, 14.27, 57.88}%, respectively.
4. A strong linear correlation exists between the reported descriptive and quantile statistics for TOR < 70 with LBGI, and TOR > 180 with HBGI.
5. The mean J_index and GMI for is 31.6 and 6.47, respectively.

**Table 1.** Summarized Statistics for Glucose Variability Within the n = 75 OPEN Data Set.

	Mean	SD	Min	Max	Q1	Q2	Q3	IQR
Interday SD (mg/dL)	43.34	10.93	23.27	75.95	34.76	41.16	49.74	14.97
Interday CV (%)	32.45	4.99	20.41	50.23	29.42	32.27	35.29	6.05
SD ROC (mg/dL/min)	1.29	0.27	0.76	2.29	1.08	1.29	1.44	0.51
TOR < 70 (%)	3.66	2.50	0.05	14.67	2.00	2.96	5.47	3.83
TIR (%)	82.08	10.35	42.07	96.90	76.94	85.93	89.82	12.89
TOR > 180 (%)	14.27	11.03	0.83	57.88	5.77	10.27	19.28	12.81
J_index	31.60	10.49	15.95	70.37	23.86	27.96	36.81	14.14
LBGI	1.07	0.57	0.06	3.26	0.69	0.94	1.51	0.78
HBGI	3.34	2.39	0.38	12.74	1.60	2.42	4.32	3.06
GMI	6.47	0.43	5.77	7.94	6.17	6.39	6.71	0.53

Abbreviations: CV, coefficient of variation; GMI, glucose management index; HBGI, high blood glucose index; IQR, interquartile range; LBGI, low blood glucose index; SD ROC, standard deviation of ROC; TIR, time-in-range; TOR, time-out-of-range.

### Clustering Glucose Profiles

Like within the OpenAPS Data Commons,^9^ we found no distinct clusters of glucose profiles within the OPEN data set. This, along with the above outcomes, adds further evidence that OS-AID systems are not flawed in achieving glucose regulation and does not cause additional harm.

### Glucose Variability Analysis Based on Gender

We divided the OPEN data set by males (n = 47) and females (n = 27) and calculated glucose variability metrics with respect to individuals’ self-reported gender. Table 2 summarizes the gender-wise descriptive statistics for each metric, as well as the results of statistical tests for distribution analysis. Supplementary Figure 6 displays distributions of demographic metrics including age, height, and weight, as well as glucose analysis metrics including TOR < 70, TIR, TOR > 180, and the SD ROC for glucose profiles, by gender. Supplementary Figure 7 shows the distribution plots for the calculated GV metrics based on gender (n = 47 males, n = 27 females).

**Table 2.** Glycemic Variability Metrics by Gender (47 Males [M], 27 Females [F]) Within the n = 75 OPEN Data Set.

	Mean [F]	Mean [M]	SD [F]	SD [M]	Min [F]	Min [M]	Max [F]	Max [M]	Skewness [F]	Skewness [M]	*P* value (z-test)	*P* value (KS)	*P* value (MW)
Interday SD (mg/dL)	47.07	41.06	11.14	10.26	32.12	23.27	75.95	70.66	0.84	0.57	**<.05**	.06	**<.05**
Interday CV (%)	34.72	31.07	4.79	4.62	27.48	20.41	50.23	41.43	1.29	−0.03	**<.05**	**<.05**	**<.05**
SD ROC (mg/dL/min)	1.44	1.20	0.24	0.25	0.98	0.76	2.29	1.81	1.59	0.32	**<.05**	**<.05**	**<.05**
TOR **<** 70 (%)	4.65	3.09	2.85	2.11	0.57	0.05	14.67	8.82	1.42	0.73	**<.05**	.06	**<.05**
TIR (%)	78.91	84.00	10.12	10.11	48.43	42.07	91.72	96.90	−1.17	−1.71	**<.05**	**<.05**	**<.05**
TOR > 180 (%)	16.44	12.91	11.33	10.74	2.94	0.83	50.38	57.88	1.18	1.77	.19	.31	.07
J_index	33.81	30.24	11.37	9.81	19.68	15.95	70.37	59.96	1.30	1.09	.16	.50	.07
LBGI	1.24	0.97	0.61	0.52	0.27	0.06	3.26	2.10	0.99	0.51	**<.05**	**<.05**	**<.05**
HBGI	3.82	3.05	2.61	2.23	0.97	0.38	12.74	11.48	1.58	1.60	.18	.50	.07
GMI	6.53	6.44	0.46	0.42	5.82	5.77	7.94	7.89	1.11	0.99	.40	.72	.22

Abbreviations: CV, coefficient of variation; GMI, glucose management index; HBGI, high blood glucose index; IQR, interquartile range; KS, Kolmogorov-Smirnov; LBGI, low blood glucose index; MW, Mann-Whitney; SD ROC, standard deviation of ROC; TIR, time-in-range; TOR, time-out-of-range.

In the context of a statistical tests performed, the outcome p<0.05 means that the observed difference or effect is statistically significant at the 0.05 level.

Salient observations from the GV outcomes by gender include the following:

1. TIR mean ± SD in males is 84.00 ± 10.11 and in females is 78.91 ± 10.12. For males, the mean ± SD for TOR < 70 is 3.09 ± 2.11 and for TOR > 180 is 12.91 ± 10.74. For females, TOR < 70 is 4.65 ± 2.85 and TOR > 180 is 16.44 ± 11.33.
2. The minimum, mean, and maximum LBGI for females and males are {0.27, 1.24, 3.26} and {0.06, 0.97, 2.10}, respectively. Similarly, the minimum, mean, and maximum HBGI for males and females is {0.38, 3.05, 11.48} and {0.97, 3.82, 12.74}, respectively. The LBGI and HBGI distributions for males and females are positively skewed. Multiple statistical tests show that there is a significant difference between the male and female distributions for LBGI (*P* < .05), but in the case of HBGI the distributions are similar (*P* > .05).
3. The minimum, mean, and maximum GMI for males and females is {5.77, 6.44, 7.89} and {5.82, 6.53, 7.94}, respectively. Both male and female distributions are positively skewed (females: 1.11, males: 0.99), and the reported statistical tests suggest a similarity between the two distributions.
4. J_index distributions follow similar patterns with positive skewness with mean values at 31.81 and 30.24 for females and males, respectively.
5. There is a statistically significant difference between female and male distributions for SD ROC (*P* < .05). The average SD ROC mg/dL per minute is 1.44 and 1.22 for females and males, respectively.
6. For TOR > 180, a similarity between female and male distributions is found across all statistical tests (*P* > .05) with a skewness of 1.18 and 1.77. For TIR, a statistically significant difference is found across all statistical tests and a negative skewness of −1.17 and −1.71. For TOR < 70, the skewness is 1.42 for females and 0.73 for males. The z-test and Mann-Whitney (MW) U-test report *P* < .05, while the Kolmogorov-Smirnov (KS) test reports with *P* = .064.
7. The average Interday CV and SD among females are 47.07 mg/dL and 34.72%, and for males are 41.06 mg/dL and 31.07%.

#### Time series Analysis for Glucose Data Based on Gender

**Figure 2.**
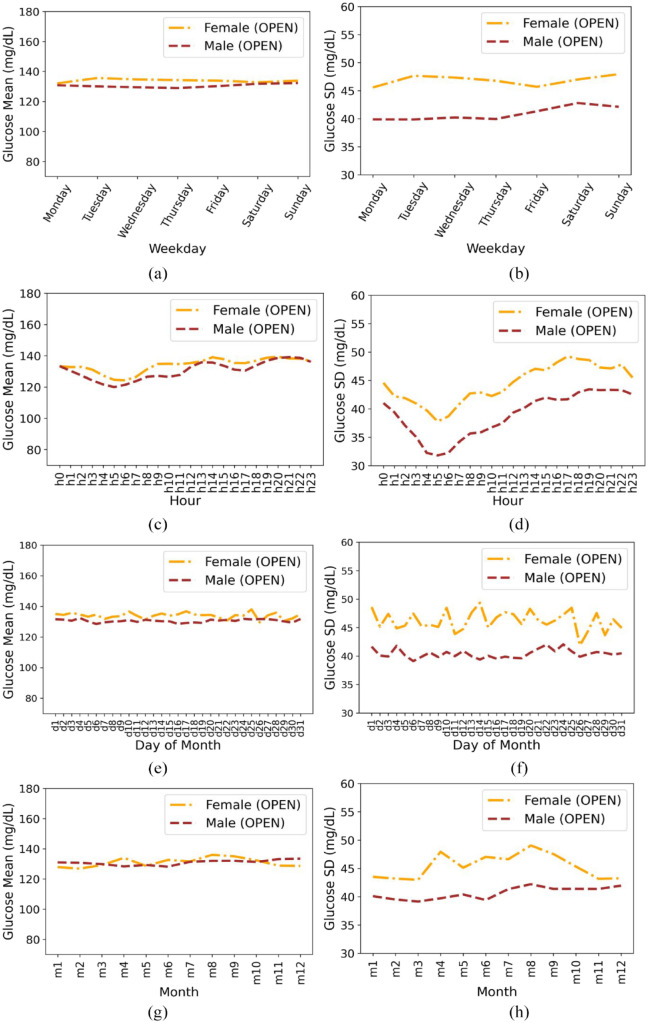
Based on gender for insulin-requiring individuals in the OPEN data set: Average glucose mean (a) and standard deviation (b) during days of the week; Average glucose mean (c) and standard deviation (d) during hours of day; Average glucose mean (e) and standard deviation (f) during days of a month; Average glucose mean (g) and standard deviation (h) during months of the year. Total number of males and females is 47 and 27, respectively.

This section presents the results of a time-series analysis of the variation in glucose mean and SD across different time periods (hours of a day, days of the week and month, and months of the year) for individuals in the OPEN data set, illustrated in Figure 2. We also analyze the average trends and differences between males and females in terms of glucose mean and SD (in mg/dL).

We found the following:

- The overall mean ± SD for females and males is 134.48 ± 47.07 and 130.75 ± 41.06, respectively.
- Both males and females follow similar trends in terms of mean glucose, with males having slightly lower values than females. The minimum mean glucose for females is 132.10 on Monday and the minimum mean glucose for males is 128.92 on Thursday. The SD is slightly higher for females throughout the week than males.
- Both females and males show a minimum in mean and SD in the morning hours.
- Females have a slight uptick in mean glucose in the morning compared to males. Males have an uptick around midday and then a slight increase across the evening hours (highest mean at 9 pm with 139.05). Females have their highest mean at 8 pm with 139.28. The SD is higher for females (minimum 37.78, maximum 49.21) than males (minimum 31.80, maximum 43.43) throughout the day.
- The glucose mean is again lower in males throughout the month as compared to females and higher SD is observed for females than males throughout the month.

### Comparative Analysis of Results Between Two Rich OS-AID Data Sets

This section compares between the n = 122 version of the OpenAPS Data Commons (46 070 days of glucose data with an average 377 days per participant, n = 50 males and n = 28 females)^9^ and the n = 75 OPEN data set of open-source AID users (36 827 days with an average of 491 days for each participant, n = 47 males and n = 27 females).

**Figure 3.**
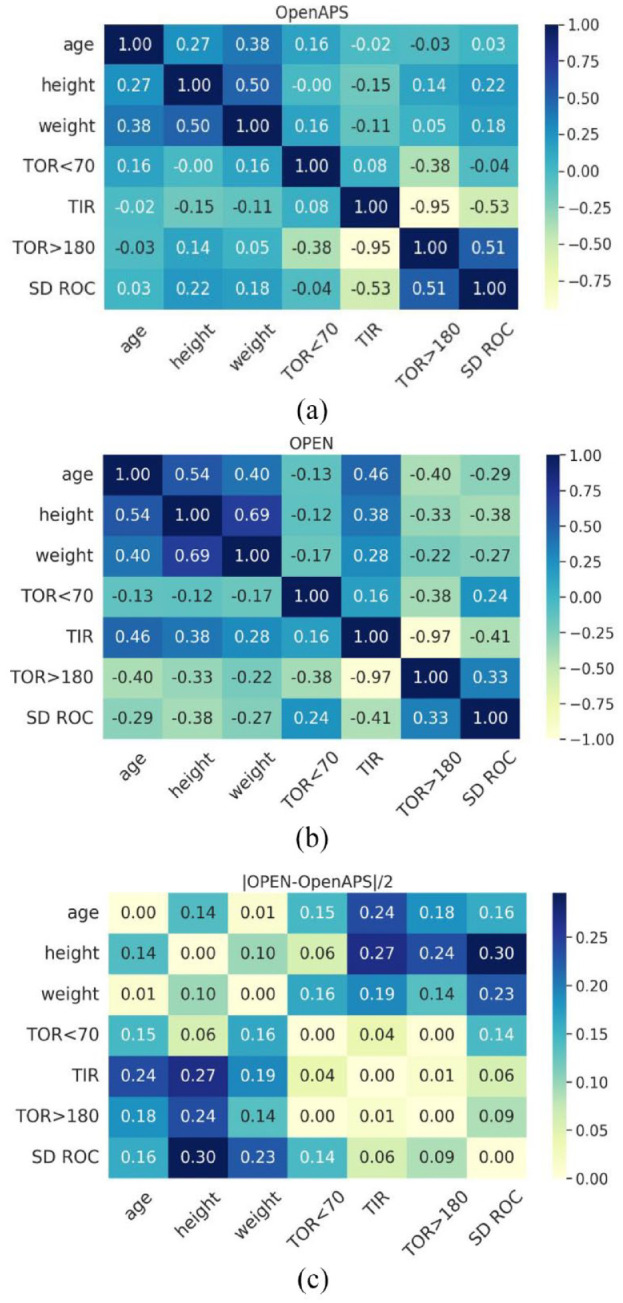
A heatmap of the correlation matrix between demographic features of the (a) n = 122 version of the OpenAPS Data Commons and (b) OPEN n = 75 data set. (c) Absolute difference between the correlation matrixes of OPEN and OpenAPS Data Commons. Abbreviations: SD ROC, standard deviation of ROC; TIR, time-in-range; TOR, time-out-of-range.

Figure 3 shows a heatmap with correlation matrix between demographic features (age, height, and weight) and glucose analysis metrics (TOR < 70, TIR, TOR > 180, and SD ROC) between both data sets. The light color approaching −1 indicates a negative linear correlation, while a dark color approaching 1 indicates a positive linear correlation.

Although there are no strong positive correlations between the demographics and glucose analysis metrics, TIR is strongly anti-correlated with TOR > 180 for both data sets. Similarly, SD ROC is slightly correlated with TOR > 180 (OpenAPS Data Commons: 0.51; OPEN: 0.33). The correlations among glucose analysis metrics are consistent among themselves across both data sets as is illustrated by small absolute differences except for TOR < 70 with SD ROC. Supplementary Figure 8 compares the distributions of SD ROC for both data sets.

### Glucose Variability Analysis Between Two Data Sets

After calculating the GV metrics including HBGI, LBGI, GMI, J_Index, CV, SD, TIR, and TOR > 70 and TOR < 180 for the OPEN data set (Supplementary Table 2), we plotted the overall distribution of GV outcomes for both data sets as illustrated in Supplementary Figure 9. Salient observations include the following:

- All GV metrics, except for TOR < 70 and LBGI, show a statistically significant difference among the underlying distributions for both data sets with *P* < .05 for z-test, KS-test, and MW-test.
- The minimum, mean, and maximum LBGI for the OpenAPS and OPEN data sets are {0.13, 1.09, 3.82} and {0.06, 1.07, 3.26}, respectively. Similarly, the minimum, mean, and maximum HBGI for the OpenAPS and OPEN data sets are {0.03, 4.36, 13.25} and {0.38, 3.34, 12.74}, respectively. The LBGI and HBGI distributions for both data sets are positively skewed. There is a statistically significant difference between the data set distributions for HBGI (*P* < .05), but LBGI distributions are similar (*P* > .05).
- The minimum, mean, and maximum GMI for the OpenAPS and OPEN data sets are {5.40, 6.63, 7.96} and {5.77, 6.47, 7.94}, respectively. Both distributions are skewed (OpenAPS: −0.11; OPEN: 1.04 with *P* < .05).
- J_index distributions follow similar patterns with positive skewness, where the OPEN data set has a higher skewness score (1.22), with respective mean values of 36.42 and 31.60 for the OpenAPS and OPEN data sets.
- The average SD ROC mg/dL per minute is 1.42 and 1.29 for the OpenAPS and OPEN data sets, respectively.

## Discussion

This paper assessed glycemic variability of the second-largest data set of individuals using open-source AID systems. The analysis was performed on the OPEN data set (n = 75) and compared with the previously studied version of the OpenAPS Data Commons (n = 122).^9^ The OPEN data set presents an extensive data set, providing an additional 36 827 days worth of glucose data for analysis with an average of 491 days per participant, and the combined data sets have 82 897 days-worth of glucose data.

In the OPEN data set, the mean TIR was found to be 82.08%. The TOR < 70 and TOR > 180 were 3.66% and 14.3%, respectively. The gender-based analyses revealed similar TIR. A significant proportion of the participants in the OPEN data set achieved a TIR (>70%), TOR < 70 (<4%), and TOR > 180 (<25%) better than the recommended standards for people with insulin-requiring diabetes.^16^

The results indicate that while there is an inverse relationship between TIR and TOR > 180, there is little correlation between TOR < 70 and TIR. It is plausible that because TOR < 70 is low relative to the average for most PwD (the majority of whom are unable to access AID), the relative effect on TIR is thus muted compared to those who may have TOR < 70 far from goal levels for PwD; this finding may be unique to AID users and should be further evaluated.

We observed that there are no significant gender differences within the data sets. For example, the glucose mean and SD by day of the week is lower in males than in females in both data sets. In both data sets, the SD was lowest around morning hours (5-6 am). There are more similarities (eg, above-standard TIR glucose outcomes) than differences (slightly differing patterns in male versus female glucose mean and SD for months of the year) between the data sets.

The work for this paper—creating deduplication methods to check for duplicate individuals present in different data sets—is particularly important. It allows a growing pool of data for additional analyses in diabetes. Additionally, this method can be applied to other large-scale data sets of many types of data and multiple variables, enabling researchers to work across data sets gathered by different groups and/or donated by different pools of individuals. This could be valuable for researchers assessing other donated, anonymized real-world data sets where participants may have altruistically provided data to multiple research data sets.

### Limitations

There are a few limitations in this work, including those previously discussed^9^ regarding time series and gender-based analyses performed on different lengths of data. Specific to these two data sets, there are some differences in how data was collected from the OPEN survey compared the OpenAPS Data Commons demographics, and limitations regarding the OPEN data set have been previously described at length elsewhere.^17^ Additional work leveraging comparisons or combinations of these data sets should continue to pay close attention to the cleaning and use of these variables in analyses. Some of the variances between gender observed in these analyses could be explained by other factors, and future studies that have the benefit of prospectively designed data collection should collect additional variables (such as different insulin types used, among other factors such as CGM type), which was not available in this data set due to the retrospective nature of the study.

### Future Research

The above-goal TIR and low TOR < 70 previously shown in the OpenAPS Data Commons^9^ as well as within this analysis of the OPEN data set of open-source AID users should not only be taken as an indicator of what is currently being achieved in the “DIY” (do-it-yourself) community; it is a reflection of the continued progress of open-source AID systems in producing improvements to the quality of life of PwD.^17-22^

There are many machine learning tools that could be applied to these data sets. While this paper primarily focused on glucose variability and gender time series, this data set alongside the OpenAPS Data Commons also contains data with carbohydrate entries, insulin dosing data, and algorithm-derived variables. This rich repository of user behaviors (carbohydrate entries/meal estimates; target adjustments; exercise information) as well as algorithmic-derived variables (such as insulin sensitivity fluctuations) and dosing decisions within this data set will be useful for additional applied research for creating benchmarks and applying improvements to open-source and commercial AID systems in the future.

## Conclusion

The previously developed open-source analysis framework^9^ for evaluating glycemic variability has been applied to two large data sets of open-source AID users. A deduplication procedure has been developed and applied prior to a combined analysis of the two data sets, using clinically approved standard glucose variability metrics. Both the OPEN and OpenAPS Data Commons data sets show TOR < 70, TIR (70-180), and TOR > 180 within recommended goal levels, adding additional evidence of real-world efficacy of OS-AID. This study provided further evidence of the real-world efficacy of open-source AID and paved the way for further research into the relationships between individual user behaviors and glycemic variability outcomes.

## Supplemental Material

sj-docx-1-dst-10.1177_19322968231198871 – Supplemental material for Glucose Variability Analysis in Two Large-Scale and Real-World Data Sets of Open-Source Automated Insulin Delivery Systems Supplemental material, sj-docx-1-dst-10.1177_19322968231198871 for Glucose Variability Analysis in Two Large-Scale and Real-World Data Sets of Open-Source Automated Insulin Delivery Systems by Drew Cooper, Bernd Reinhold, Arsalan Shahid and Dana M. Lewis in Journal of Diabetes Science and Technology
