# Supplementary material for: Glucose Variability Analysis in Two Large-Scale and Real-World Data Sets of Open-Source Automated Insulin Delivery Systems
Source: J Diabetes Sci Technol. 2023 Sep 26;19(3):649–57. doi: 10.1177/19322968231198871 (PMC12035276; doi:10.1177/19322968231198871)
Supplement: sj-docx-1-dst-10.1177_19322968231198871 – Supplemental material for Glucose Variability Analysis in Two Large-Scale and Real-World Data Sets of Open-Source Automated Insulin Delivery Systems [file sj-docx-1-dst-10.1177_19322968231198871.docx]

Supplemental Appendix

**Glucose Variability Analysis in Two Large-Scale
and Real-World Datasets of Open-Source
Automated Insulin Delivery Systems**

**Drew Cooper, Bernd Reinhold, Arsalan Shahid, and Dana M. Lewis**


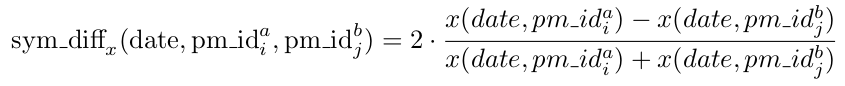


*Supplementary Figure 1: The formula for defining the symmetric difference of a statistical momentum (mean, SD, min, max, count),
a, b indicates the two datasets and i(j) represent a pm_id in the dataset a(b).

We first computed mean, standard deviation, minimum, maximum and count (referred to collectively as “statistical momenta”) of sensor glucose values for each day in both datasets. Symmetric difference (sym_diff) is then calculated between the two datasets for each momentum.*

*If the symmetric difference is below 1% for all five momenta, it indicates a high degree of matching in the glucose data for a day between individuals in both datasets. We would therefore conclude these data represent the same individual in both datasets.*

*
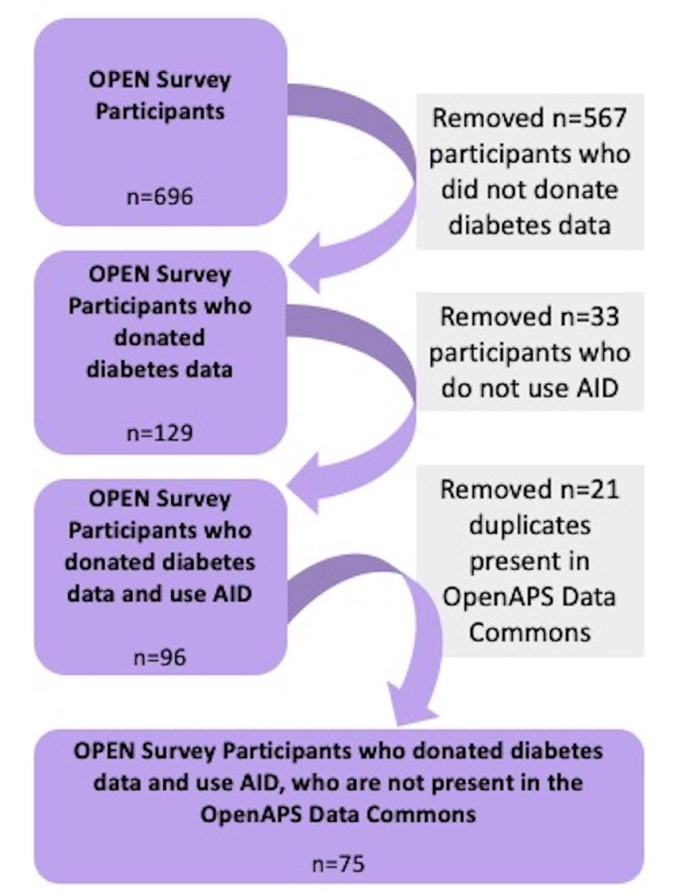
*

*Supplementary Figure 2: A flow diagram illustrating the process of removing non-AID users, duplicates between the two datasets, and final cleaning checks to ensure the n=75 remaining dataset had AID data present.*

Supplementary Table 1. Count of Demographics Data for OPEN dataset

| **Demographic Features** | **Available Reports** | **Missing Reports** |
| --- | --- | --- |
| Total Number of Individuals | 75 | 0 |
| Diagnosed Date | 73 | 2 |
| Date of Pump Use | 72 | 3 |
| Date of CGM Use | 74 | 1 |
| Date of Closed Loop Initiation | 74 | 1 |
| AID Type | 74 | 1 |
| DOB | 74 | 1 |
| Country | 74 | 1 |
| Weight | 71 | 4 |
| Height | 72 | 3 |
| Last A1c | 61 | 14 |
| Last A1c Date | 60 | 15 |
| Gender | 74 | 1 |


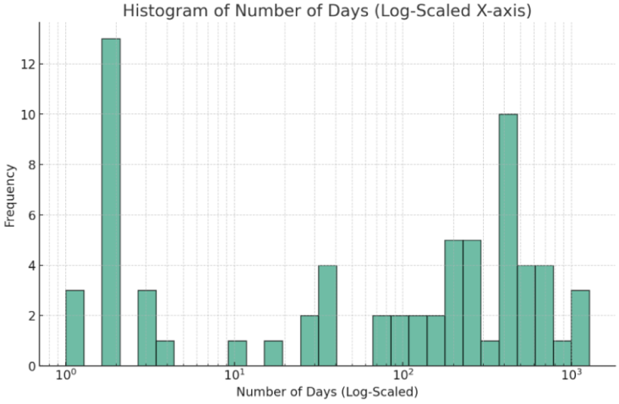


*Supplementary Figure 3: A histogram with a log-scaled x-axis provides a view of the distribution of days per participant with the data set, given that the data spans several orders of magnitude. There are several clusters of participants with different lengths of data; such as around 10 days; between 100 and 200 days; and around 1000 days.*


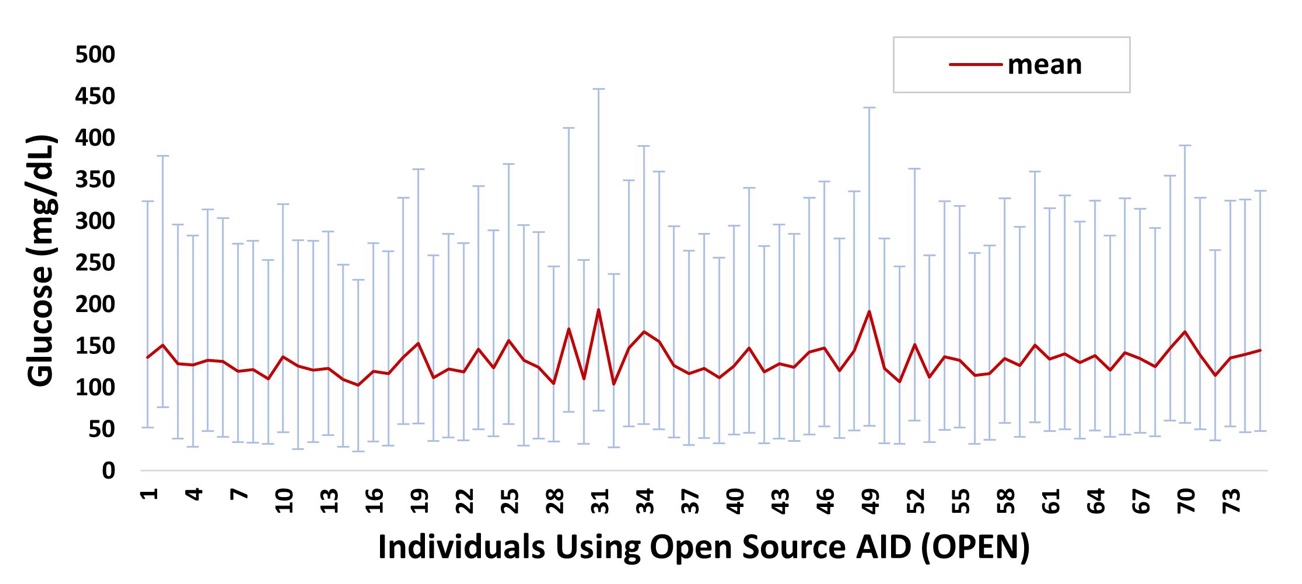


*Supplementary Figure 4: Glucose mean and distribution for insulin-requiring individuals using open-source AID systems. Total number of individuals (n) = 75. Average glucose mean is 132.20 mg/dL and SD across the individuals is 43.34 mg/dL.*


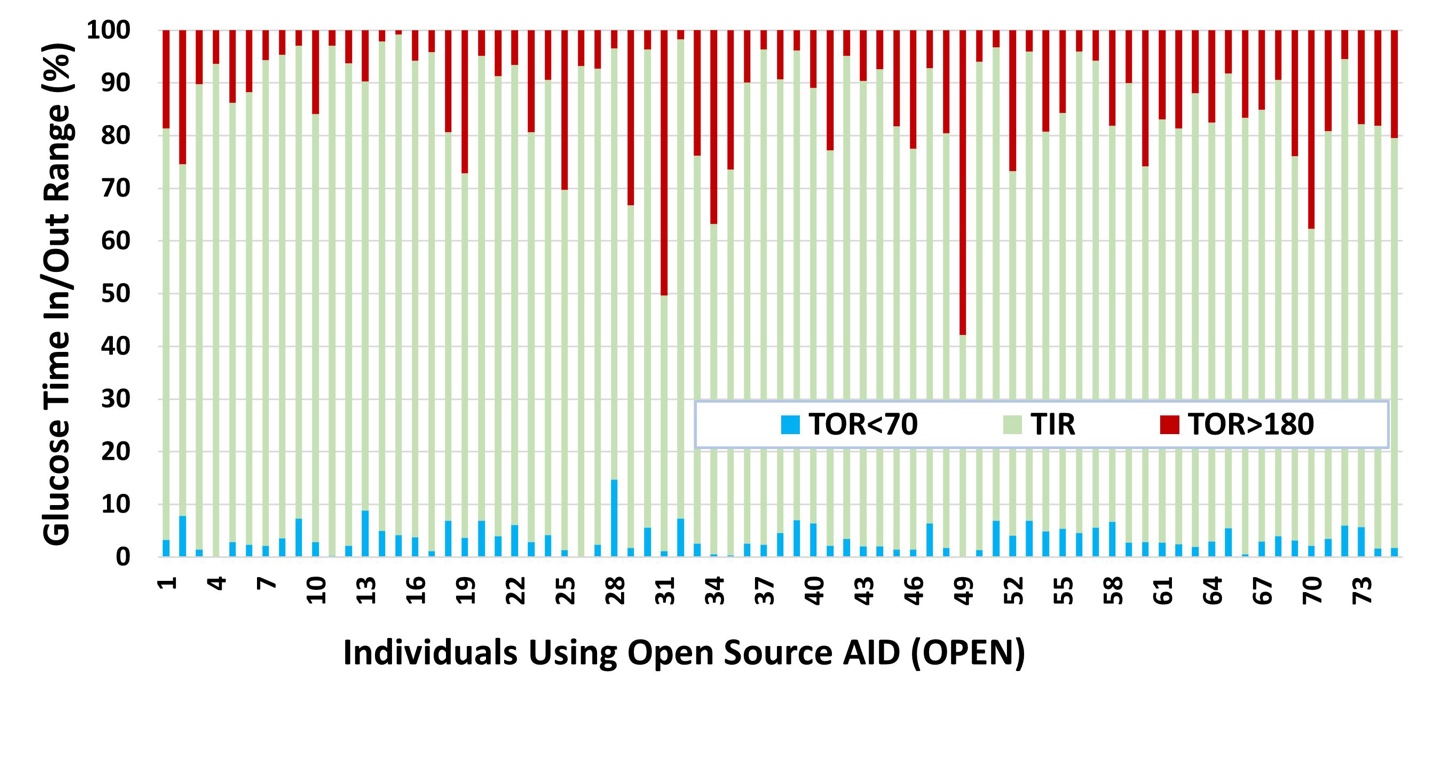


*Supplementary Figure 5: Glucose TIR and TOR for insulin-requiring individuals using open-source AID systems. Total number of individuals (n) = 75. The average TIR (TOR<70, TOR>180) for insulin-requiring individuals in the OPEN datasets is 82.08%, (3.66%, 14.3%).*


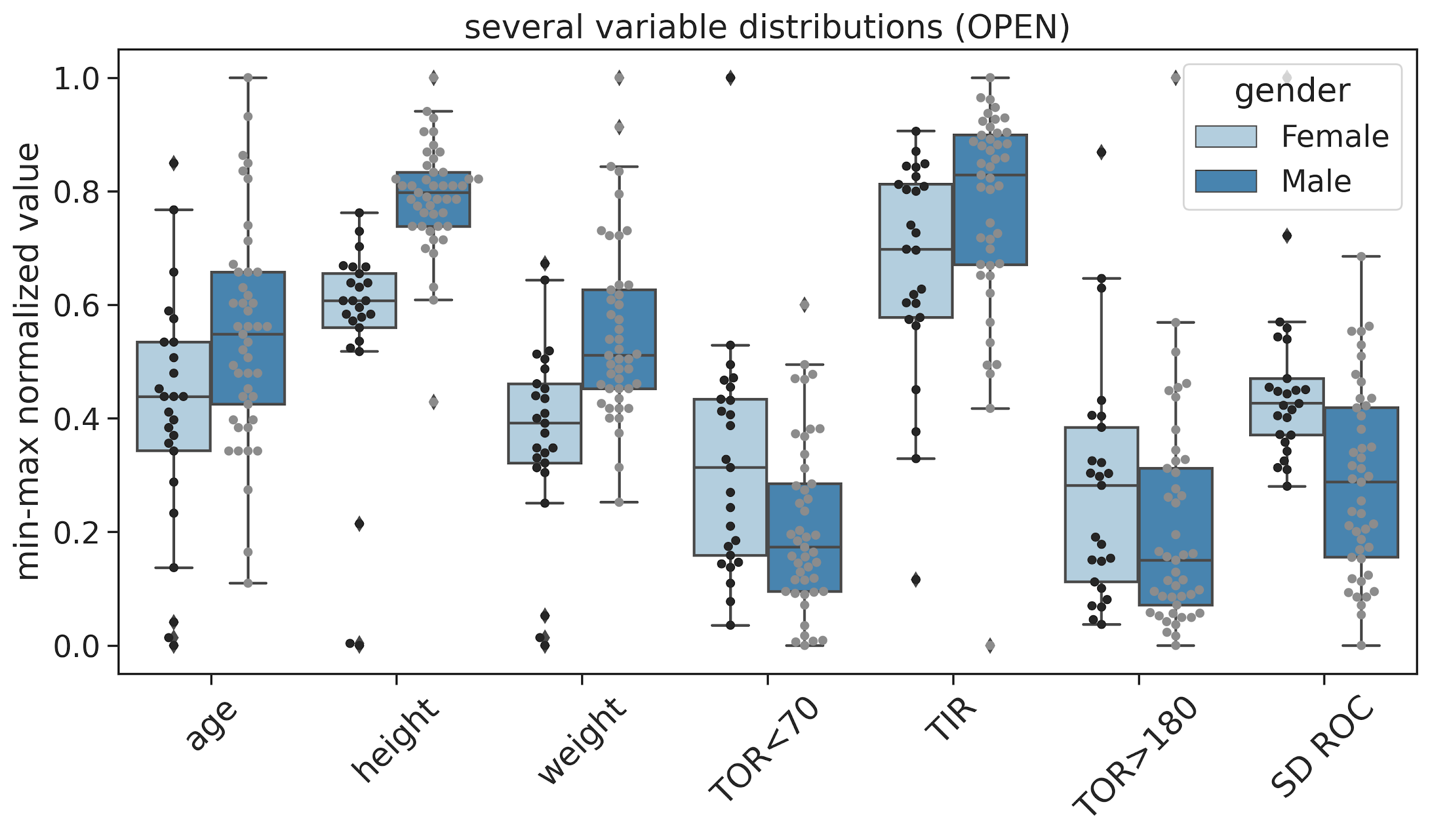


*Supplementary Figure 6: Gender-wise distributions of age, height, weight, TOR<70, TIR, TOR>180, and standard deviation glucose rate of change (SD ROC). The age, height, and weight were reported by 21 and 43 females and males, respectively. Other glucose analysis metrics are reported for 27 females and 47 males.*

| 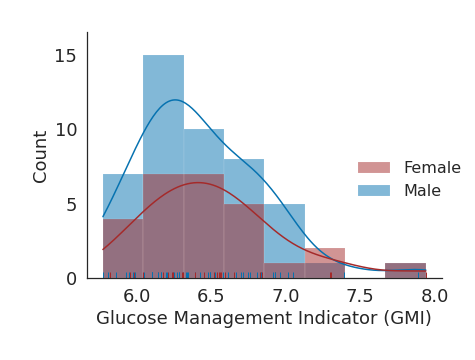 | 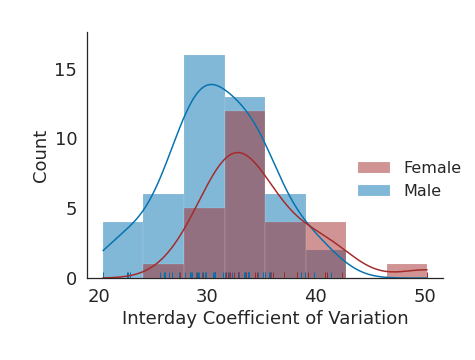 **(B)** |
| --- | --- |
| 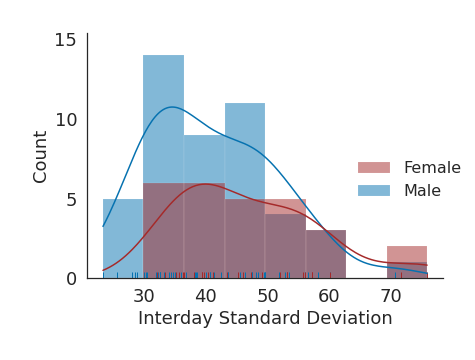  **(C)** | 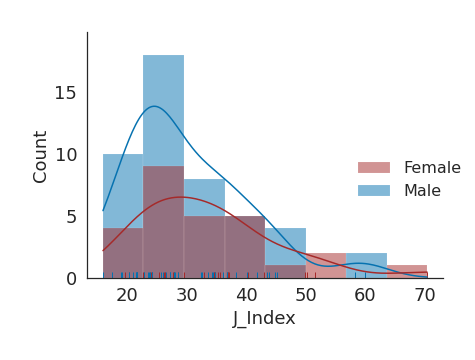  **(D)** |
| 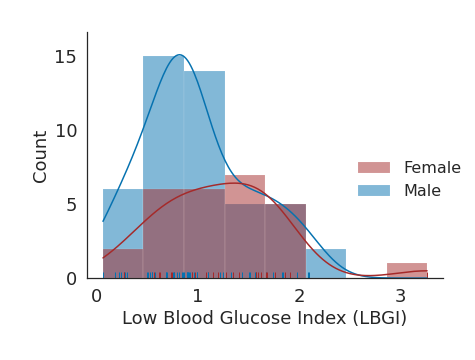  **(E)** | 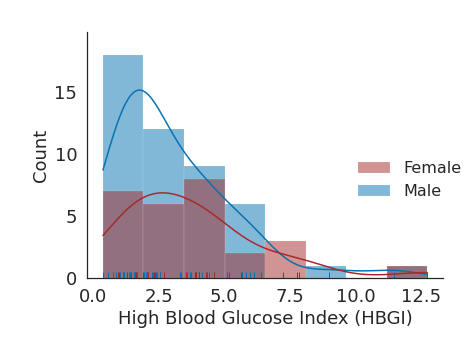  **(F)** |
| 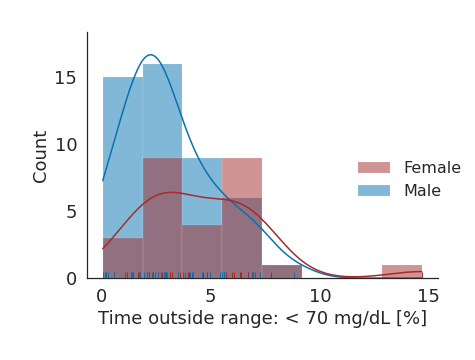  **(G)** | 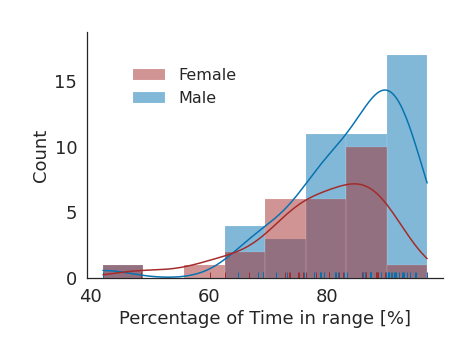  **(H)** |
| 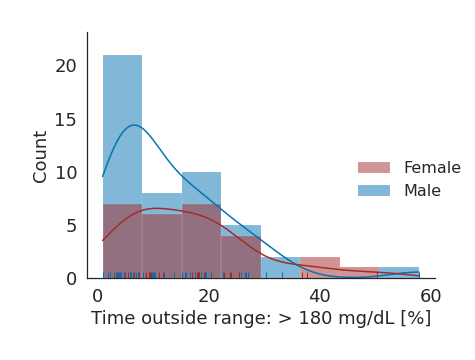  **(I)** | |

*Supplementary Figure 7: Glucose variability outcomes for individuals using open-source AID systems based on gender. Total number of males and females is 47 and 27, respectively. (A) GMI (B) CV. (C) SD. (D) J_index. (E) LBGI. (F) HBGI. (G) TOR<70. (H) TIR. (I) TOR>180.*


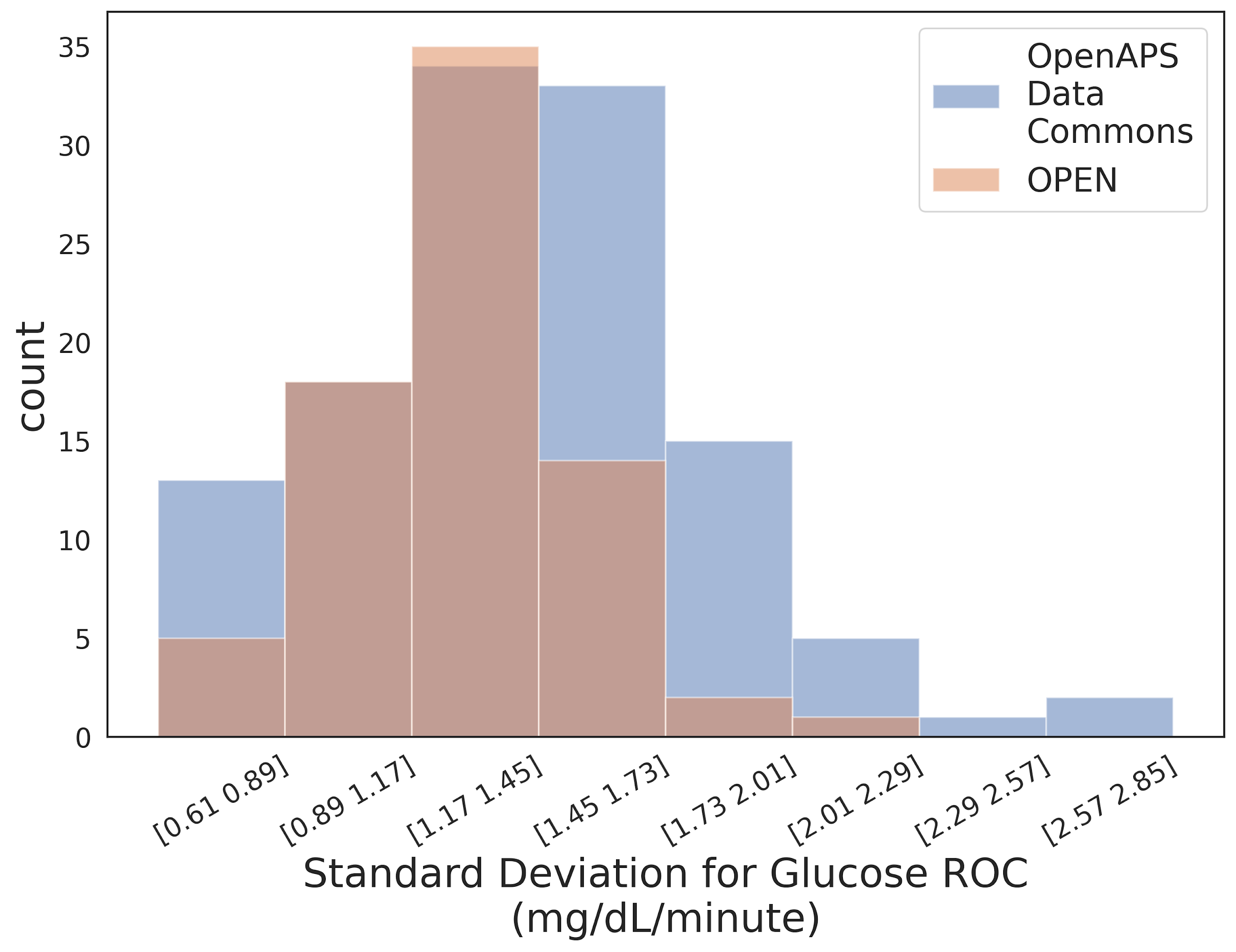


*Supplementary Figure 8: Standard deviation of glucose rate of change (SD ROC) for the OPEN dataset (n=75) and the OpenAPS Data Commons dataset (n=122). The skewness score for OpenAPS Data Commons and OPEN datasets is 0.48 and 0.54, respectively. The Shapiro-Wilk test follows the skewness scores with p<0.05 confirming the underlying distributions are non-normal. The mean±SD for OPEN and OpenAPS Data Commons is 1.29±0.27 and 1.42±0.39, respectively. This may be an artifact of earlier (more 2015–2018) data and the comparison to the n=122 version of OpenAPS Data Commons versus a more recent version of the dataset.*

Supplementary Table 2: Comparison of statistical measures of the OpenAPS Data Commons and OPEN datasets. Z-test determines whether the means of two distributions are significantly different, KS is the Kolmogorov-Smirnov test, MW the Mann-Whitney U test.

|  | Mean [OpenAPS] | Mean [OPEN] | SD [OpenAPS] | SD [OPEN] | Min [OpenAPS] | Min [OPEN] | Max [OpenAPS] | Max [OPEN] | Skewness [OpenAPS] | Skewness [OPEN] | p-value (z-test) | p-value  (KS) | p-value (MW) |
| --- | --- | --- | --- | --- | --- | --- | --- | --- | --- | --- | --- | --- | --- |
| Interday SD (mg/dL) | 49.75 | 43.34 | 11.22 | 10.93 | 14.71 | 23.27 | 77.33 | 75.95 | -0.38 | 0.66 | **<0.05** | **<0.05** | **<0.05** |
| Interday CV (%) | 35.43 | 32.45 | 5.04 | 4.99 | 16.86 | 20.41 | 44.94 | 50.23 | -0.84 | 0.43 | **<0.05** | **<0.05** | **<0.05** |
| SD ROC (mg/dL/min) | 1.42 | 1.29 | 0.39 | 0.27 | 0.61 | 0.76 | 2.69 | 2.29 | 0.48 | 0.54 | **<0.05** | **<0.05** | **<0.05** |
| TOR<70 (%) | 4.01 | 3.66 | 2.96 | 2.50 | 0.23 | 0.05 | 16.97 | 14.67 | 1.52 | 1.30 | 0.40 | 0.72 | 0.34 |
| TIR (%) | 77.26 | 82.08 | 8.95 | 10.35 | 49.75 | 42.07 | 98.45 | 96.90 | -0.13 | -1.39 | **<0.05** | **<0.05** | **<0.05** |
| TOR>180 (%) | 18.74 | 14.27 | 9.77 | 11.03 | 0.05 | 0.83 | 49.67 | 57.88 | 0.25 | 1.49 | **<0.05** | **<0.05** | **<0.05** |
| J_index | 36.42 | 31.60 | 10.48 | 10.49 | 10.39 | 15.95 | 73.93 | 70.37 | 0.26 | 1.22 | **<0.05** | **<0.05** | **<0.05** |
| LBGI | 1.09 | 1.07 | 0.65 | 0.57 | 0.13 | 0.06 | 3.82 | 3.26 | 1.57 | 0.82 | 0.84 | 0.87 | 0.36 |
| HBGI | 4.36 | 3.34 | 2.28 | 2.39 | 0.03 | 0.38 | 13.25 | 12.74 | 0.59 | 1.62 | **<0.05** | **<0.05** | **<0.05** |
| GMI | 6.63 | 6.47 | 0.43 | 0.43 | 5.40 | 5.77 | 7.96 | 7.94 | -0.11 | 1.04 | **<0.05** | **<0.05** | **<0.05** |


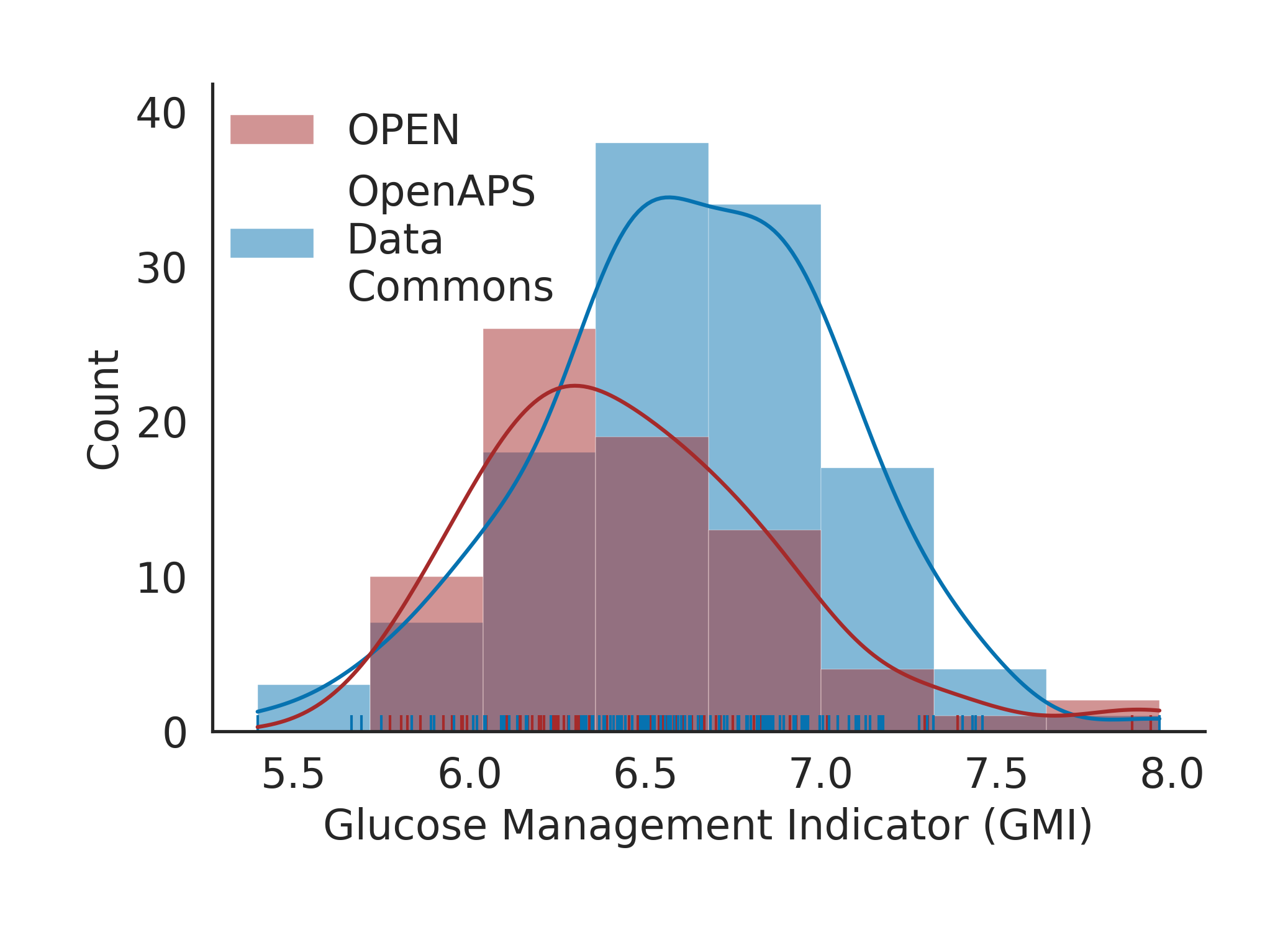

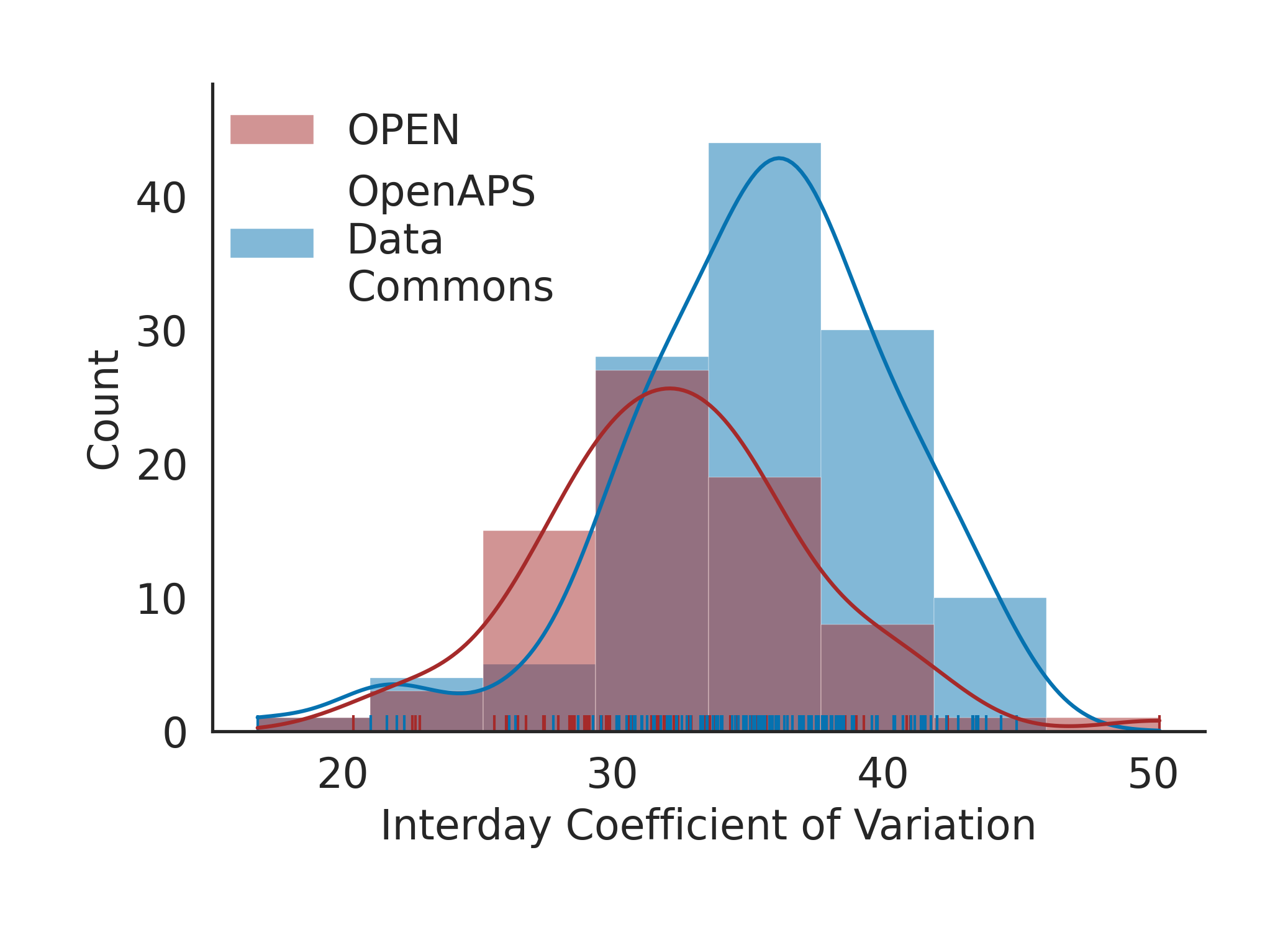


| **(A)** | **(B)** |
| --- | --- |
| 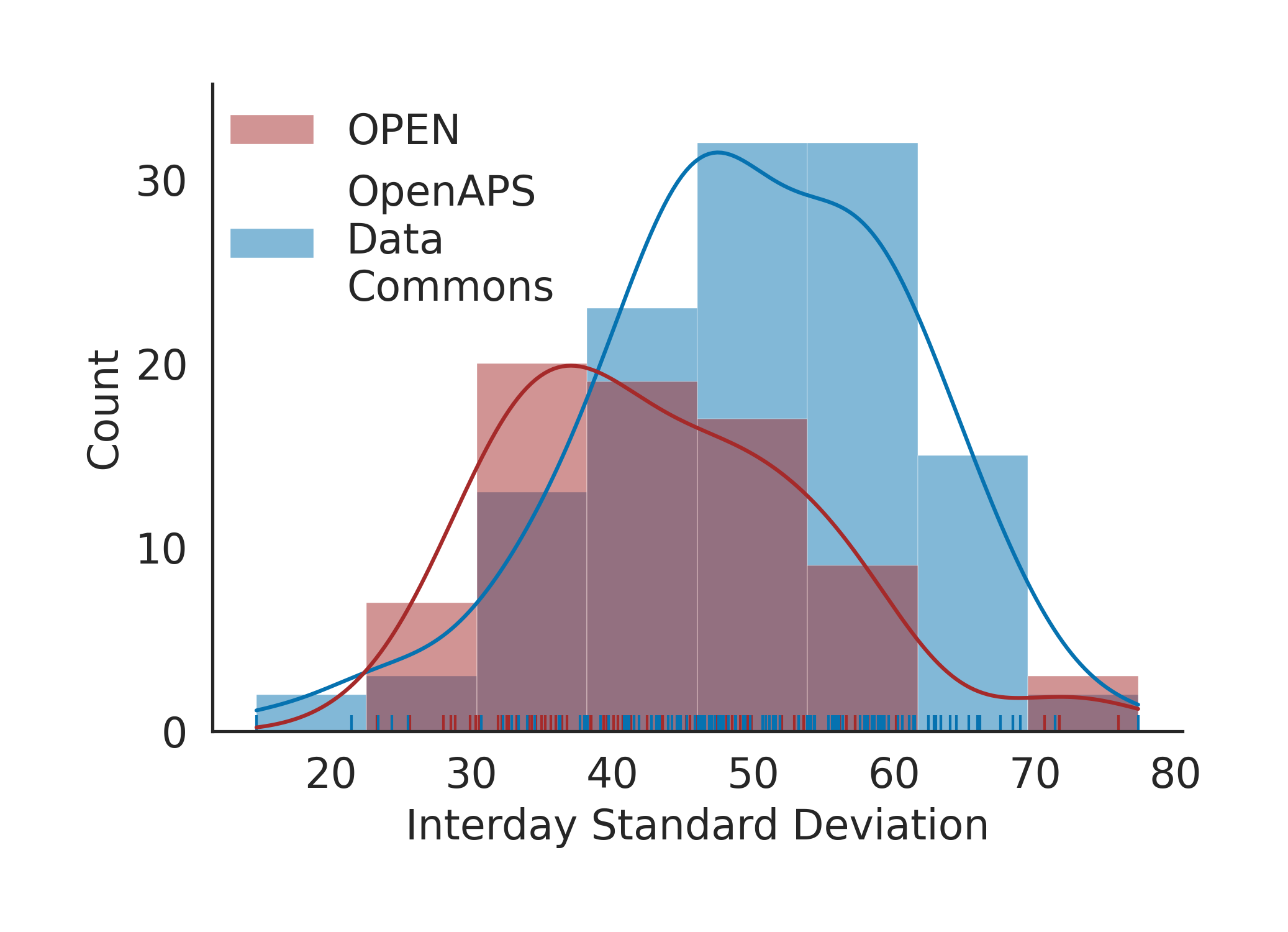  **(C)** | 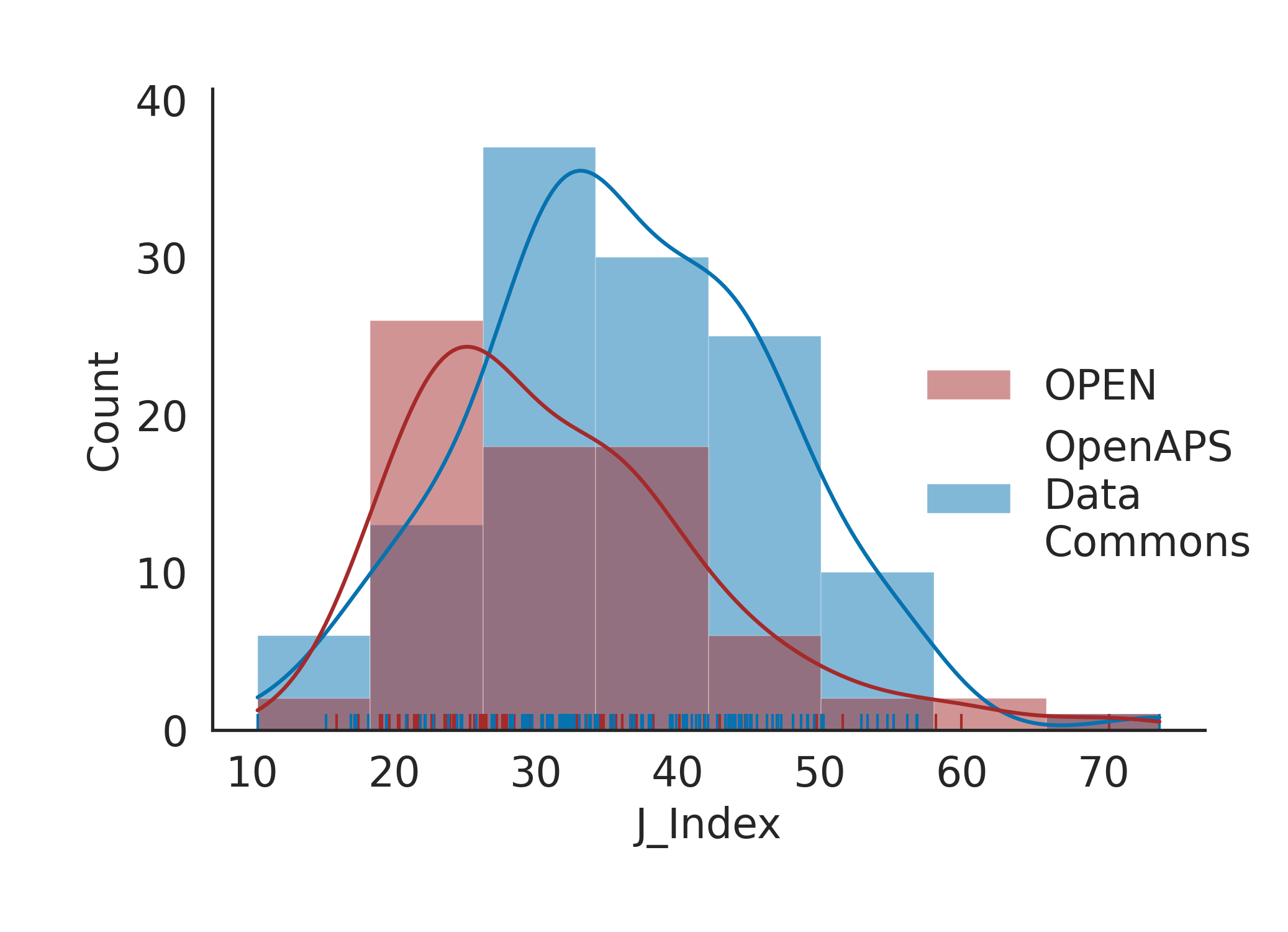  **(D)** |
| 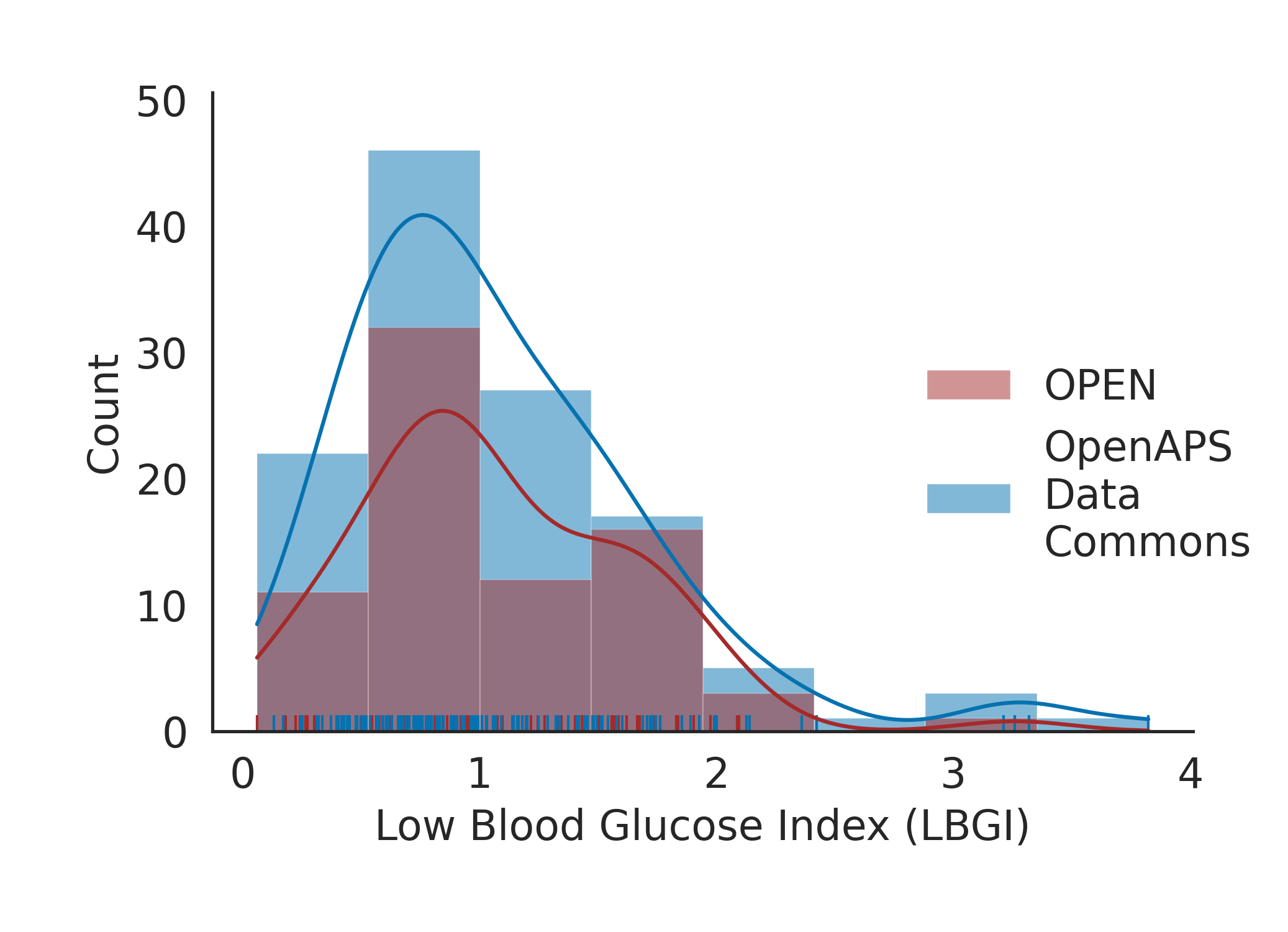  **(E)** | 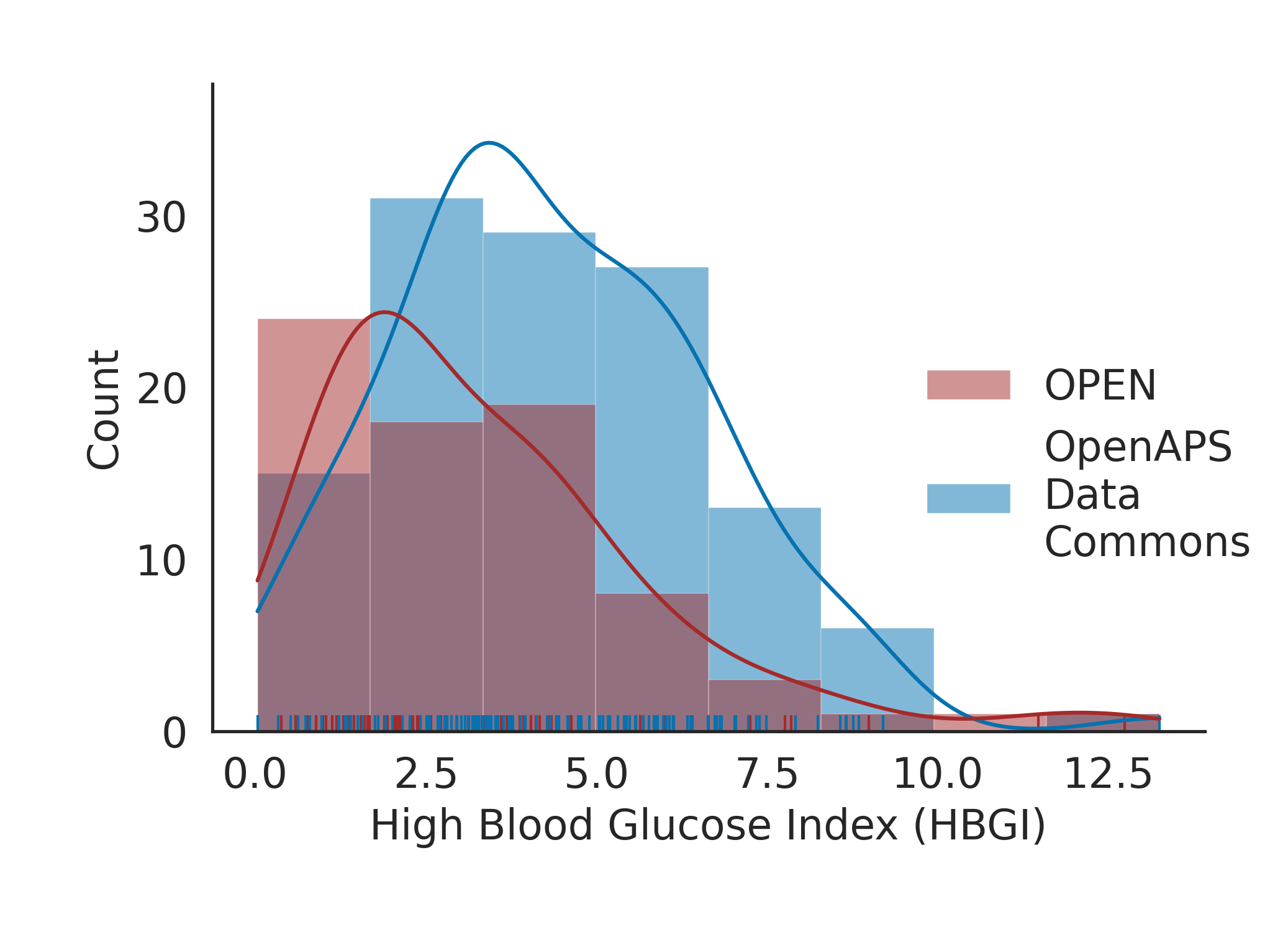  **(F)** |
| 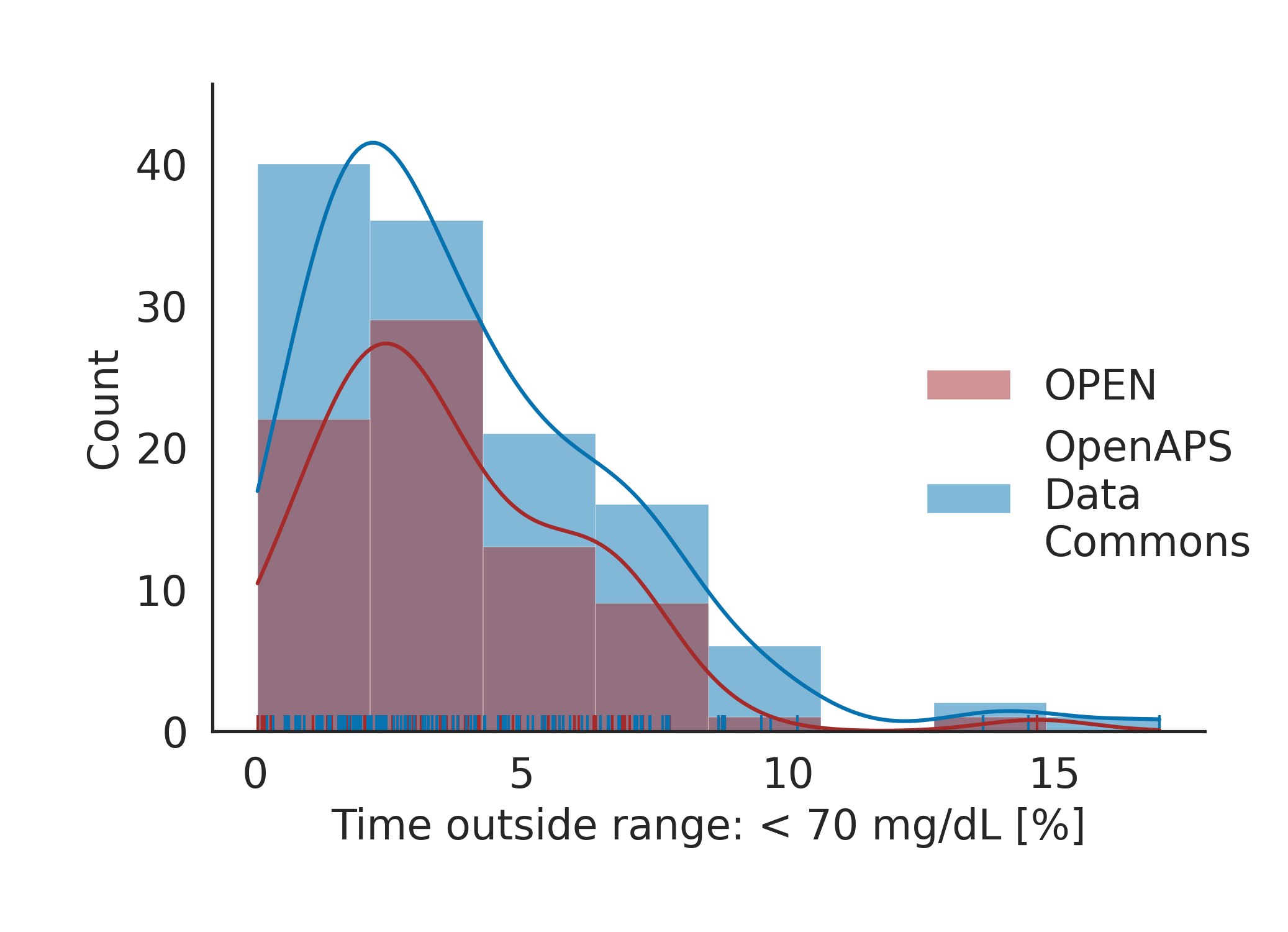  **(G)** | 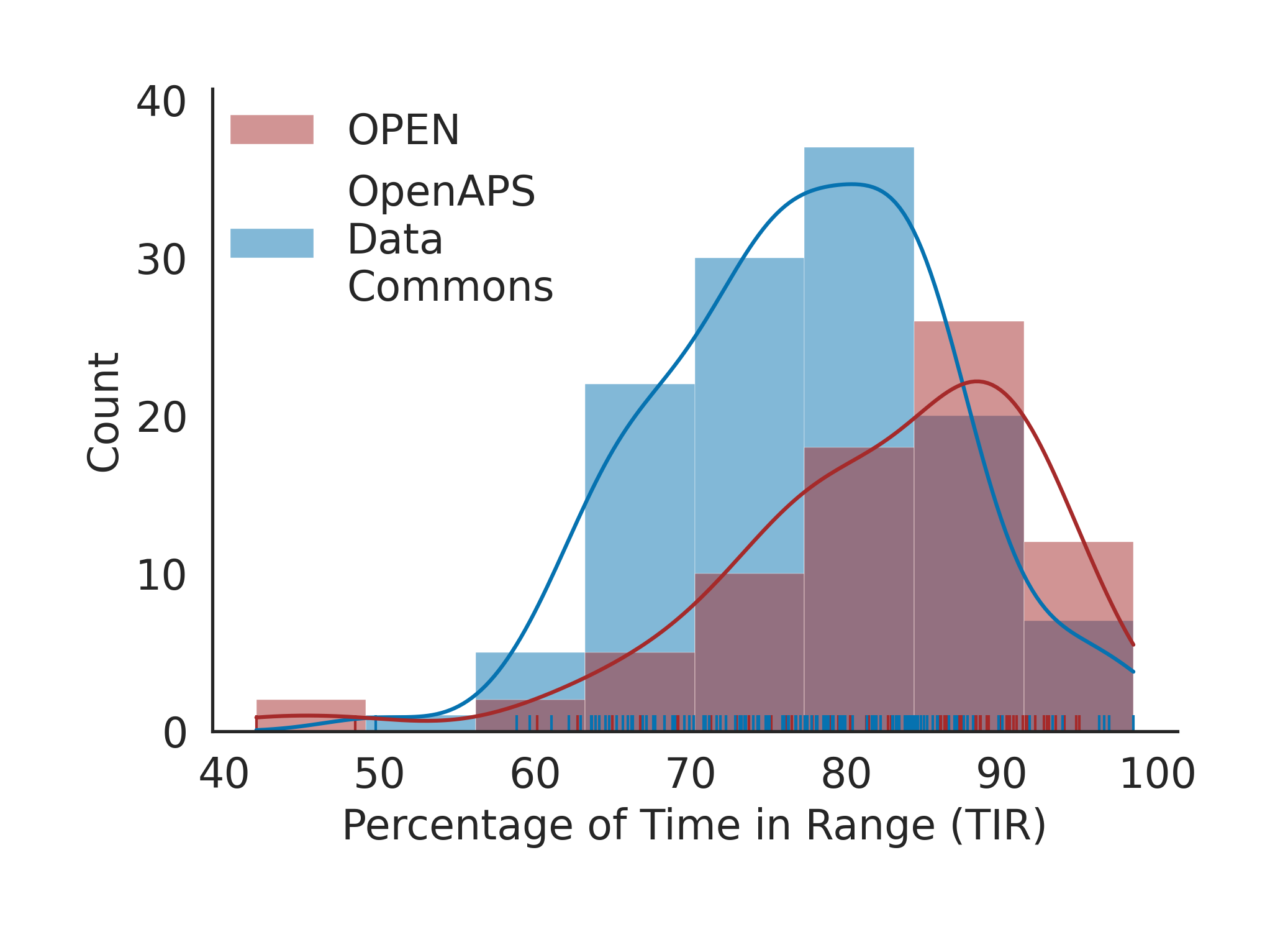  **(H)** |
| 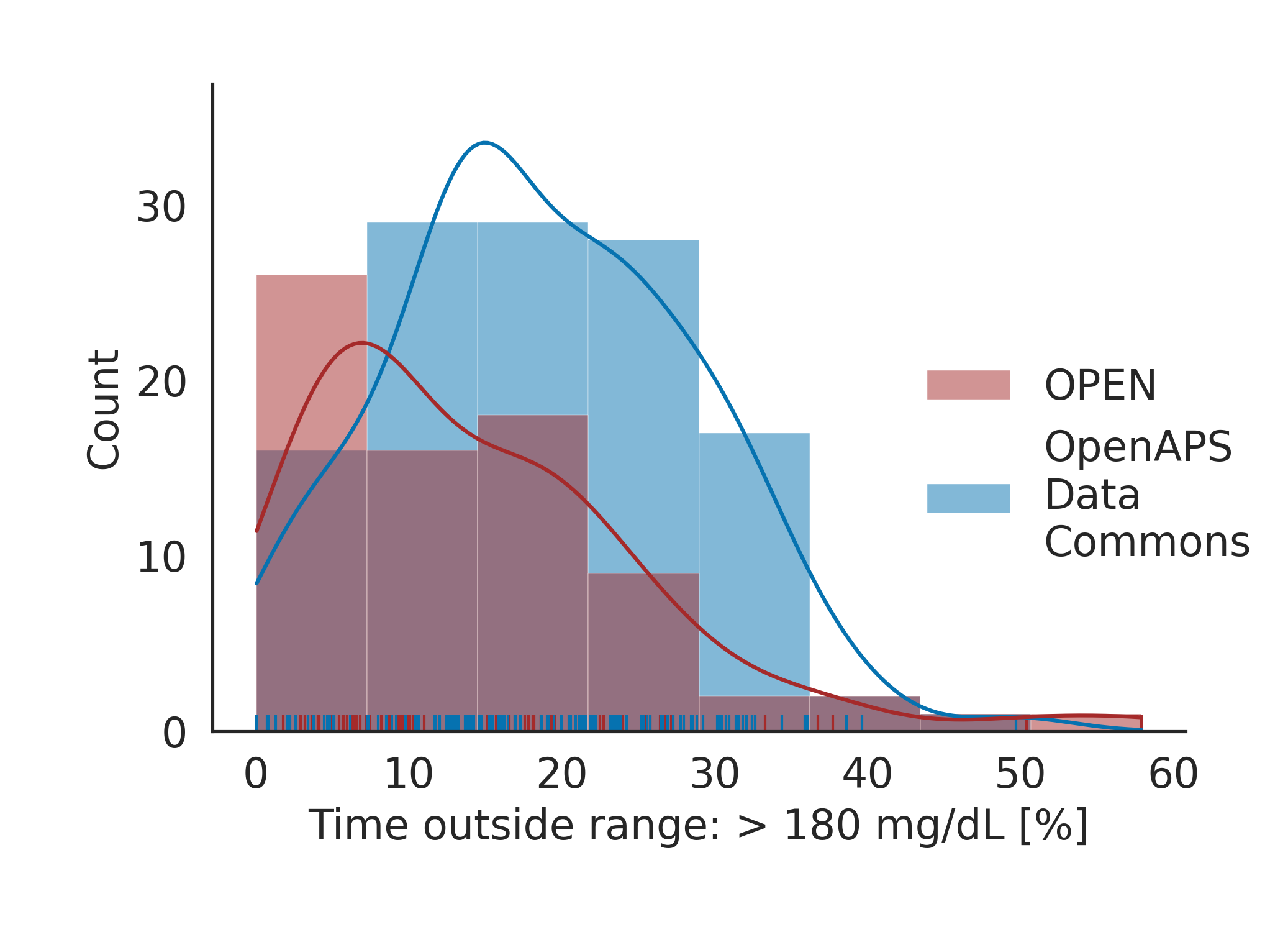  **(I)** | |

*Supplementary Figure 9: Glucose variability outcomes for individuals using open-source AID systems in each of the OPEN (n=75) and OpenAPS Data Commons (n=122) datasets. (A) GMI (B) Coefficient of Variation (CV). (C) Standard Deviation (SD). (D) J_index. (E) HBGI. (F) LBGI. (G) TOR<70. (H) TIR. (I) TOR>180*
